# The Shuar Health and Life History Project: Overview at 20 Years and Introduction to the Special Issue

**DOI:** 10.1002/ajhb.70207

**Published:** 2026-02-03

**Authors:** Samuel S. Urlacher, Theresa E. Gildner, Lawrence S. Sugiyama

**Affiliations:** ^1^ Department of Anthropology Baylor University Waco Texas USA; ^2^ Department of Anthropology Washington University in St. Louis St. Louis Missouri USA; ^3^ Department of Anthropology University of Oregon Eugene Oregon USA

**Keywords:** Amazon, behavioral ecology, evolutionary medicine, evolutionary psychology, global health, human biology, indigenous health

## Abstract

The Shuar Health and Life History Project (established in 2005) is an interdisciplinary, integrated field and laboratory research project with the Indigenous Shuar population in Amazonian Ecuador. Grounded in human biology, behavioral ecology, evolutionary psychology, evolutionary medicine, and global health, the SHLHP has three key research foci: (1) To identify how market integration (via effects on diet, pathogen exposure, lifestyle, etc.) impacts Shuar health and well‐being; (2) To investigate (using evolutionary life history theory) how lifetime phenotype and health are shaped by adaptive energy allocation between competing life tasks; and (3) To test hypothesized human psychological and demographic adaptations, including aspects of sociality that are central to the evolutionary success of our species. To address these foci, the SHLHP has established long‐term and mutually beneficial relationships with the Shuar and local collaborators, resulting in community‐engaged data collection with more than 3500 participants and a wide range of research publications and policy contributions over the past 20 years. This special issue of the *American Journal of Human Biology* showcases 10 original SHLHP articles that span much of the project's intellectual breadth and represent important advances for understanding human biology, life history, and health. To serve as an introduction, here we provide essential background on the Shuar and the SHLHP, overview the ten included special issue articles, and discuss key research and impact goals for the next 20 years of the SHLHP.

## 1 Introduction

This special issue of the *American Journal of Human Biology* highlights the research and intellectual contributions of the Shuar Health and Life History Project (SHLHP, established in 2005; https://www.shuarproject.org). This is a personal topic for the issue's guest editors and authors of the present paper, who represent SHLHP co‐directors (SSU, LSS), the founder (LSS), and a senior researcher (TEG). In this introduction, we have three objectives: First, to provide a brief background on the Shuar and the SHLHP, including the SHLHP's research foci and prior contributions to human biology and related disciplines. Second, to present an overview of the 10 SHLHP original research articles that are included in this special issue, indicating how each can advance our understanding of human biology, life history, and health. Third, to provide concluding remarks about the special issue and point to the next 20 years of the SHLHP. Readers seeking information on specific project topics or a more in‐depth reflection on the long‐term operation of the SHLHP are referred to focused project publications in this special issue and elsewhere (see below).

## 2 Shuar and the SHLHP

### 2.1 Shuar: A Brief Background

A wealth of information on the Shuar has been published over the past century by ethnographers, ecologists, linguists, historians, political scientists, and others (e.g., Barrett and Haley 2006; Bennett et al. 2002; de Salvador Agra and Martínez Suárez 2015; Gerique 2010; Harner 1984; Karsten 1919; Kroeger 1980; Lu 2007; Pellizaro and Náwech 2005; Rubenstein 2001; Rudel 2021; Zapata‐Ríos et al. 2009). Prior community‐engaged work by the SHLHP has also provided extensive information on Shuar ecology and recent way of life (e.g., Liebert et al. 2013; Urlacher, Blackwell, et al. 2016; Urlacher, Liebert, et al. 2016). Here, we highlight information that readers are likely to find the most critical for contextualizing the work included in the special issue.

**FIGURE 1:**
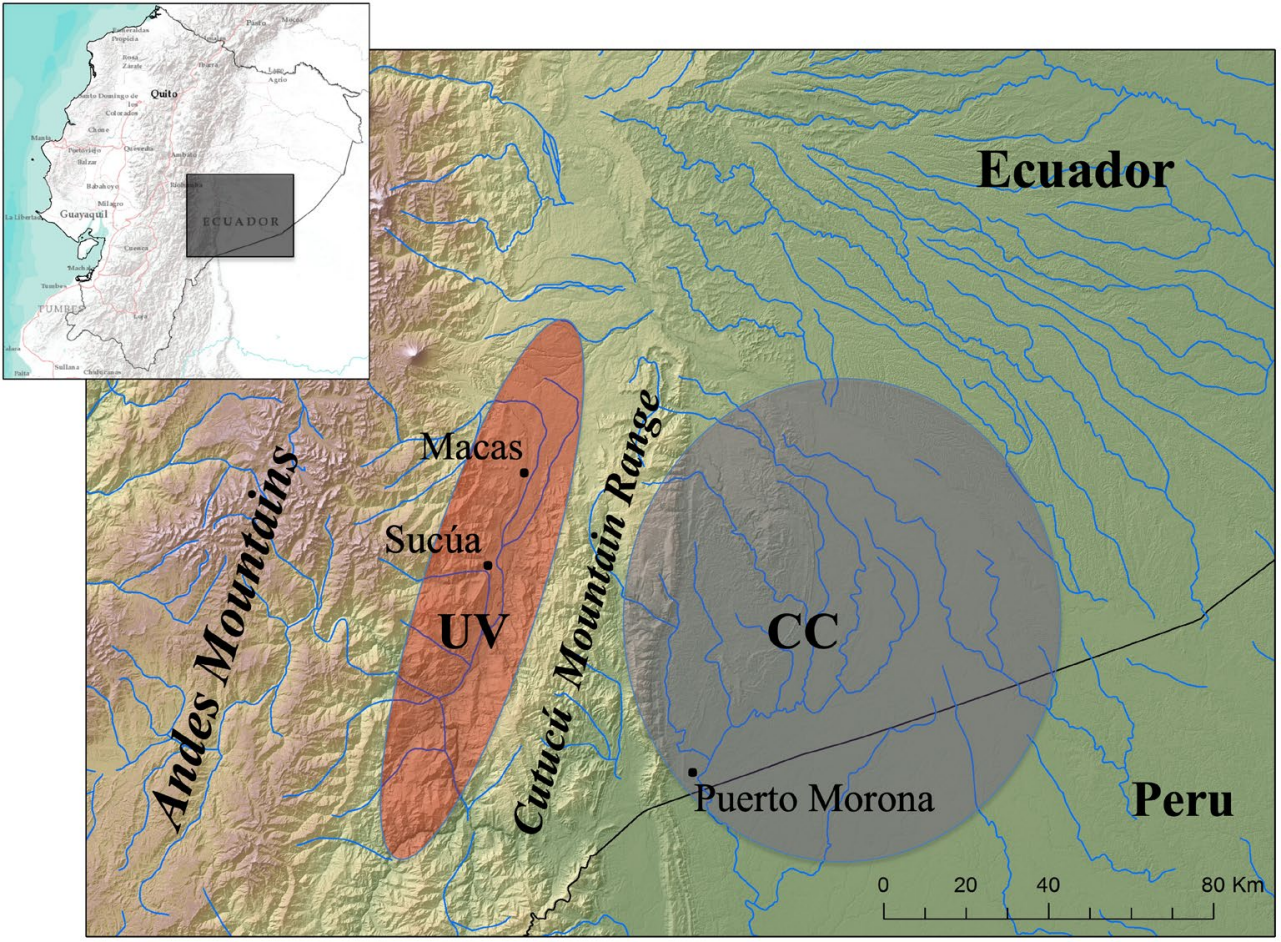
Map of Shuar territory in Ecuador and Peru. Shaded areas indicate the geographically distinct *Upano Valley* (UV, red) and *Cross‐Cutucú* (CC, blue) regions. The SHLHP has collected data with Shuar living in the UV and CC in Ecuador since 2005 (totaling more than 3500 participants from over 60 communities). Data have also been collected with neighboring non‐Indigenous Ecuadorians and Shuar who have immigrated to the United States.

The Shuar are a large Indigenous population of the Chicham (formerly Jivaroan) language group, currently numbering ≈136 000 individuals in the neotropical upper Amazonas region of Ecuador and Peru (INEC 2023). In total, their core territory spans ≈30 000 km^2^, encompassing more than 600 communities spread across two distinct geographical regions (Figure 1). The western, *Upano Valley* (UV) region of Shuar territory is bound by the Andean foothills to the west and the rugged Cutucú mountain range (elevation 2225 m) to the east. The eastern, trans‐ or *cross‐Cutucú* (CC) region lies in the Amazon Basin proper east of the Cutucú, stretching as far east as the Rio Macuma.

Written records of the Shuar date back centuries. Following a brief but violent period of subjugation and successful revolt against Spanish colonizers in the late sixteenth century (De Velasco 1841), the Shuar remained generally resistant to non‐Indigenous interaction until a small trading network was established with Ecuadorian settlers (*colonos* in Spanish, *apach* in Shuar) from the highlands in the 1890s (Harner 1984). This event increased the availability of certain manufactured items in Shuar territory, including machetes, firearms, and woven cloth (Borrini and Jaireth 2007; Rubenstein 2001). Subsequent natural resource exploration and intensified missionary activity in the area beginning in the 1940s resulted in additional contact with *colonos* and the clustering of previously dispersed individual households into centralized communities, or *centros*. Around this time (in 1964), the Shuar established the *Federación Interprovincial de Centros Shuar* (FICSH), Latin America's first Indigenous federation, to further enhance agency and address agrarian reforms targeting the development of the Ecuadorian Amazon (Salazar 1981; Rubenstein 2001). Over the ensuing decades, the progressive adoption of motorized canoes, the arrival of the Ecuadorian power grid, and the construction of reliable roads—connecting the UV to major Ecuadorian cities in the late 20th century and the CC to the UV in the early 21st century—accelerated changes for many Shuar. These events and others have resulted in a rapid demographic shift, with recent Shuar population growth rate estimates of ≈3.5% and most of the population currently under the age of 15 years old (INEC 2023; Jokisch and McSweeney 2011).

**FIGURE 2:**
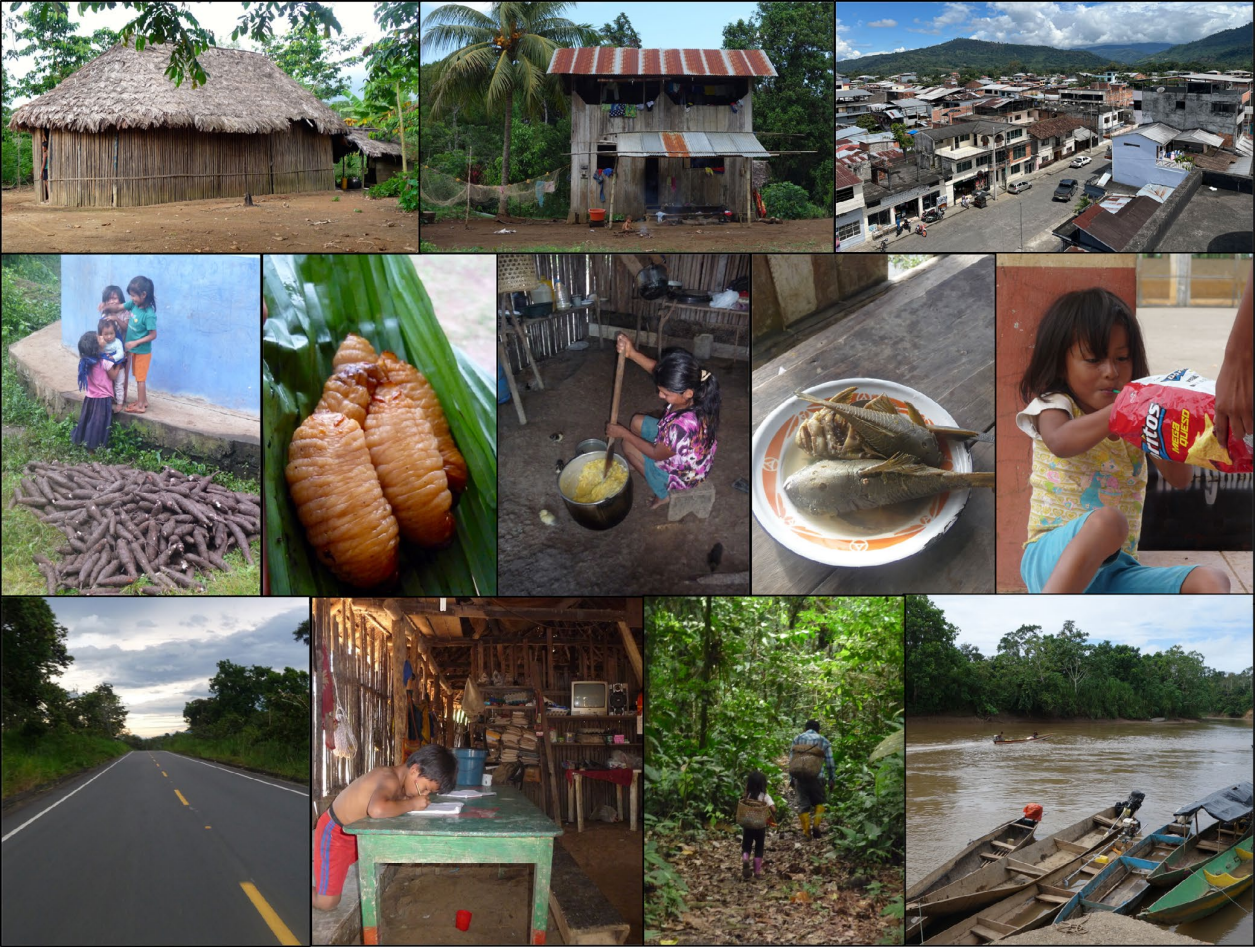
Photos illustrating variation in market integration, environment, lifestyle, and diet among the Shuar. This variation powers many SHLHP analyses. Photos by SSU.

Today, the Shuar experience wide variation in market integration—the phenomenon of increasing production for and consumption from a market‐based economy (Lu 2007)—and related environment, lifestyle, and diet (Figure 2). Many Shuar living in rural areas, particularly those in the more geographically‐ and economically‐isolated CC, continue to practice a subsistence forager‐horticulturalist lifestyle typical of Indigenous Amazonian groups (Dufour et al. 2016). This is based on swidden (i.e., “slash and burn”) horticulture, hunting, fishing, and foraging, with the primary dietary staples including sweet manioc ( *Manihot esculenta* ) and plantains ( *Musa x paradisiaca* ). Many rural communities and households remain hours of travel from market centers and without electricity, running water, and access to wage labor or Western biomedical care. At the other end of the spectrum, Shuar living in UV market centers (e.g., Sucúa or Macas, with current populations of ≈12 000 and ≈24 000, respectively) often have access to modern infrastructure and clinics/hospitals, work wage labor, and purchase the majority of their food. Notably, however, the experiences of Shuar communities and households are diverse, and most fall somewhere along the gradient between these two current market integration extremes (Gildner et al. 2020; Liebert et al. 2013; Urlacher, Liebert, et al. 2016).

### 2.2 SHLHP: Origins, Research Foci, and Intellectual Contributions

The SHLHP was founded in 2005 by LSS, with Shuar partnerships at the community level and FICSH and the Ecuadorian Ministerio de Salud as its primary collaborators. In 2007, Josh Snodgrass joined the project as co‐director. This co‐directorship was expanded to include long‐term project members SSU, Melissa Liebert, and Felicia Madimenos in 2019. Currently, the SHLHP is comprised of 14 research team members at the senior and student levels.

From the beginning, the SHLHP has prioritized long‐term community engagement, local collaboration, and participant benefit. Over the past 20 years, dozens of Ecuadorian individuals, communities, schools, organizations, and institutes have collaborated with the SHLHP, including (in addition to FICSH and the Ministerio de Salud) the Ecuadorian Ministerio de Educación and Instituto de Seguridad Social. These collaborations are essential to the ethos and functioning of the project. Among other core activities, the SHLHP strives to provide health information and capacity building to participants, community members, and other partners. These efforts include regular development of locally‐grounded health education tools and detailed research reports for local policy stakeholders. They are designed to address Shuar interests and concerns, to improve Shuar general well‐being, and to effectively respond to on‐the‐ground public health challenges. Readers are referred elsewhere (Snodgrass et al. Forthcoming) for a more detailed description of these engagement efforts and the mutually beneficial relationships between the SHLHP, research participants, and local collaborators.

The intellectual foundation of the SHLHP is drawn from the fields of human biology, behavioral ecology, evolutionary psychology, evolutionary medicine, and global health. The project's overall research foci and related publications are presented in Table 1. The research foci are: (1) To identify how market integration (via effects on diet, pathogen exposure, lifestyle, etc.) impacts Shuar health and well‐being; (2) To investigate (using evolutionary life history theory) how lifetime phenotype and health are shaped by adaptive energy allocation between competing life tasks; (3) To test hypothesized human psychological and demographic adaptations, including aspects of sociality that are central to the evolutionary success of our species.

**TABLE 1:** SHLHP research foci and related publications.

Foci	Topic	Publications
The impact of market integration on health & well‐being	Subadult growth & development	Blackwell et al. (2009), Urlacher, Blackwell, et al. (2016), Urlacher, Liebert, et al. (2016), Urlacher et al. (2021) and Urlacher, Snodgrass, et al. (2016)
	Infectious/parasitic disease	**Barrett et al.** (**2025**, 2022), Blackwell et al. (2016), Cepon‐Robins et al. (2014, 2019), Gildner et al. (2016, 2020)
	Obesity & cardiometabolic disease	Liebert et al. (2013), McDade et al. (2012), Shattuck‐Heidorn et al. (2021), **Tallman et al.** **(** **2025** **)**, Urlacher, Liebert, et al. (2016) and Urlacher et al. (2021)
	Intestinal health/function	Cepon‐Robins et al. (2019), Colehour et al. (2014), **Pfaff Nash et al.** **(** **Forthcoming** **)**, Stagaman et al. (2018)
	Reproduction and bone density	**Madimenos et al.** (**Forthcoming**, 2011a 2012, 2015, 2020), Stieglitz et al. (2016)
	Physical/psychosocial stress	Amir et al. (2019), **Barrett et al.** **(** **2025** **),** **Liebert et al.** **(** **2025** **),** **Tallman et al.** **(** **2025** **)**
	Oxidative stress	**Samsonov et al.** **(** **2026** **)**
	Anemia	DeLouize et al. (2022)
Life history theory & energetics (e.g., plasticity, trade‐offs)	Energy budgets	Christopher et al. (2019), Urlacher et al. (2019, 2021)
	Somatic growth	Blackwell et al. (2010), Urlacher et al. (2018, 2019, 2021)
	Reproduction	**Bribiescas et al.** **(** **2025** **),** **Madimenos et al.** (**Forthcoming**),(2011b, 2012, 2020), Ross et al. (2023)
	Immune activity	Blackwell et al. (2016, 2010, 2011), **Cepon‐Robins et al.** (**2025**, 2019, 2021), **Gildner et al.** (**2025**), Shattuck‐Heidorn et al. (2021), Urlacher et al. (2018, 2019, 2021)
	Physical activity	Madimenoset al. (2011b), Stieglitz et al. (2016); Urlacher et al. (2019, 2021)
	Oxidative stress/somatic maintenance	**Bribiescas et al.** **(** **2025** **)**
Psychological/cognitive and demographic adaptations	Facial preferences	Scott et al. (2014)
	Risk‐taking	Amir et al. (2020)
	Disgust/avoidance behavior	Cepon‐Robins et al. (2021)
	Anger & shame	Sell et al. (2017), Sznycer et al. (2018)
	Residence patterns	Walker et al. (2013)

*Note:* The current number of peer‐reviewed publications utilizing SHLHP data is 45. SHLHP work has been supported by NSF, NIH, the Leakey Foundation, the Wenner‐Gren Foundation, CIFAR, the University of Oregon, and other public and private funders. Bold = papers featured in the current special issue.

**FIGURE 3:**
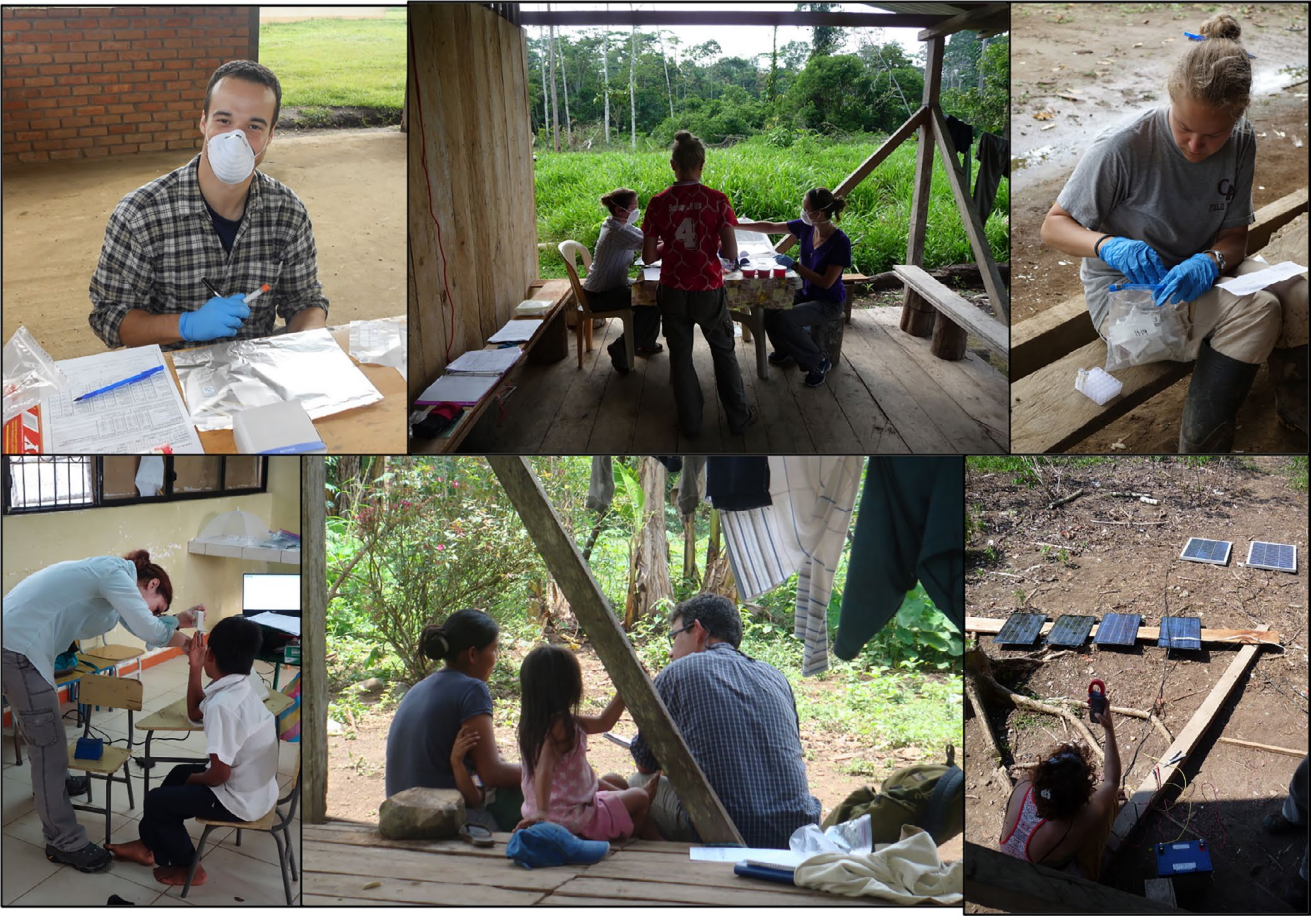
Photos of SHLHP team members collecting data and sharing results with the Shuar. The SHLHP utilizes a wide mixed‐methods toolkit, ranging from unstructured interviews to biomarker analyses. Long‐term community engagement and benefit to participants is a priority. For biospecimen collection, only minimally invasive methods are used. Photos by SSU and Melissa Liebert.

To address the project's research foci, the SHLHP has collected mixed‐longitudinal data with more than 3500 Shuar participants aged 1 month to 90 years in over 60 communities throughout the UV and CC regions of Ecuador. Data have also been collected with neighboring non‐Indigenous Ecuadorians (*colonos*) and with Shuar who have immigrated to the United States. Emphasis is placed on cultural sensitivity, ethnographically grounded (often mixed‐methods) approaches, minimally invasive collection techniques, and the integration of field‐ and lab‐based datasets (Figure 3). The current SHLHP dataset includes (among many other variables) household demographic and market integration assessments, dietary information, pathogen exposure and infection status information, reproductive and health histories, anthropometric measures, bone density information, behavior and physical activity assessments, energy expenditure measures, metabolic health metrics obtained via point‐of‐care testing, and a wide range of biomarker measures obtained via laboratory analysis of biospecimens (saliva, urine, feces, and finger‐prick dried blood spots).

Over the past 20 years, SHLHP findings have been published and shared widely, reflecting the relevance of the research not only for human biology but also for general science, behavioral ecology, evolutionary psychology, global health, evolutionary medicine, nutrition science, tropical medicine, immunology, and other disciplines. A full list of SHLHP peer‐reviewed publications (*N* = 45) and links to key media coverage are available on the project's website (https://www.shuarproject.org/). Hundreds of SHLHP conference presentations have also been made. Many of the SHLHP's studies have been powered by the wide variation in lived experience captured within the Shuar population (market integration, environment, lifestyle, diet, etc.) and have capitalized on integrated field‐ and lab‐based datasets. Some of the SHLHP's most notable intellectual contributions include: (1) Demonstrating that the effects of market integration on Shuar immune activity, child growth and development, cardiometabolic health, and other aspects of phenotype and health are heterogeneous and play out at multiple levels of analysis (i.e., geographic region, community, household, and individual) (Cepon‐Robins et al. 2014; Gildner et al. 2020; Liebert et al. 2013; Stagaman et al. 2018; Urlacher, Liebert, et al. 2016); (2) Identifying energetic trade‐offs with immune activity as an important driver of linear growth faltering among Shuar children (Blackwell et al. 2010; Urlacher et al. 2018, 2019); (3) Showing that market integration has no detectable effect on Shuar children's total energy expenditure (kcal/day), indicating a primary role of diet in driving trends in childhood obesity in low‐ and middle‐income countries (Urlacher et al. 2021, 2019); (4) Identifying the importance of early life history events (e.g., menarche) in canalizing adult bone density phenotype and osteoporosis risk (Madimenos et al. 2012); and (5) Providing evidence that the disgust emotion among humans evolved and functions flexibly via environmental cues to regulate pathogen avoidance (Cepon‐Robins et al. 2021).

The SHLHP is unique in many respects, but it has also drawn inspiration from, influenced, and directly collaborated with a number of other long‐term human biology research projects among Indigenous and subsistence‐based groups. The broad human evolutionary approach of the SHLHP and its aim to understand ecological and lifeway change can trace its development to pioneering projects such as the Kalahari Research Project (Lee 1978), the Ituri Project (Bailey and DeVore 1989), and similar early work in long‐term research among the Ache (Hill and Hurtado 2017) and Hadza (Blurton Jones, Blurton Jones 2016). In 2005, when the SHLHP began, the Tsimane Health and Life History Project in Bolivia (Gurven et al. 2017) best matched its broad scope and emphasis on mixed‐methods approaches and biomarker integration. Other projects, while aligned with the SHLHP in many ways, differed with respect to characteristics such as their emphasis on survey/panel data (e.g., the Tsimane Amazonian Panel Study, the Cebu Longitudinal Health and Nutrition Survey), limited integration of biomarker information (e.g., the Hadza Project), or data collection targeted to only a single or few communities (e.g., the Chaco Area Reproductive Ecology Project). Over the past decade, several new long‐term human biology research projects which share attributes with the SHLHP have been started, including the Orang Asli Health and Lifeways Project (Wallace et al. 2022), the Daasanach Health and Life History Project (Rosinger et al. 2024), and the Turkana Health and Genomics Project (Lea et al. 2021). These are exciting advances for the field which promise to provide additional comparative and complementary data for the SHLHP.

## 3 Papers in the Special Issue

The present special issue includes a collection of ten original articles from the SHLHP that span many of the project's research foci/topics (see Table 1) and advance its prior contributions. It is recommended that readers begin with the article provided by Snodgrass et al. (Forthcoming). This work provides an extended background on the SHLHP, including additional details on the project's intellectual roots and its partnership and long‐term investment in Shuar communities. Importantly, it also uses the SHLHP as a case study to highlight and critically reflect on the many challenges and opportunities (practical, political, and ethical) associated with starting and maintaining a long‐term human biology field research project. This represents a unique contribution to the discipline, executed with an uncommon degree of transparency, and should serve as a valuable guide to anyone operating (or considering operating) a similar project. Topics covered by Snodgrass and colleagues include, among others, establishing active local collaborators, obtaining research permissions, collecting and transporting biospecimens, navigating the physical/mental strain of fieldwork, and funding and publishing collaborative field research. We believe that readers will find additional discussion on the ethics of health‐aligned human biology field projects particularly insightful. This section will help readers think through several common issues, such as navigating the “ownership” of data and how to manage a project which is one of the few sources of biomedical health‐related information and education for participants in particularly underserved/neglected study populations. Emphasis is placed on the context‐ and culture‐specific nature of these issues and the need to develop locally grounded strategies.

Highlighting the focus of the SHLHP on market integration's impact on Shuar health and well‐being, the contribution by Madimenos et al. (Forthcoming) serves as an important investigation on Indigenous fertility transition. This work capitalizes on SHLHP reproductive history data collected with 565 adult females—including both Shuar and neighboring non‐Indigenous *colonos*—living across the wide gradient of market integration in Shuar territory. Globally, ongoing demographic transition to low fertility is documented for many populations (e.g., Becker and Barro 1988; Kaplan et al. 2002; Shenk et al. 2016), with market integration and associated lifeway changes believed to play a critical role. Madimenos and colleagues' findings demonstrate the nuanced and context‐specific nature of such changes for Indigenous populations. While clear secular trends of decreasing fertility and changes to related life history patterns (e.g., age at first birth) are identified among non‐Indigenous *colono* women, no evidence is found for group‐level secular trends in Shuar reproductive patterns. Analysis of Shuar household‐level market integration data (composite style‐of‐life measures) indicates a much more complex situation. These data indicate that certain aspects of fertility transition are indeed occurring among the Shuar, but they are seemingly restricted currently to only the most market‐integrated Shuar households. Collectively, these findings highlight the nuance of market integration effects on reproduction among small‐scale populations. They also highlight key differences in the fertility transition between the Shuar and their *colono* neighbors—likely due to differences in factors such as socioeconomic marginalization, access to Western contraceptives, life history tradeoffs, and social value placed on large family size. From the public health perspective, such differences are associated with the persistence of health inequities for the Shuar and many other Indigenous populations. Madimenos and colleagues end their contribution by focusing on the need for public health initiatives to prioritize greater access to reproductive health‐care services among the Shuar—including for family planning, information, and education—while also remaining sensitive to local priorities and cultural values.

Linking the SHLHP's interest in market integration with that in evolutionary understandings of lifetime phenotype and health, Cepon‐Robins et al. (2025) use SHLHP data to provide a novel and nuanced test of the *Old Friends Hypothesis* (OFH) for explaining the alarming rise of allergy and autoimmune conditions in populations around the globe. The OFH proposes that this health crisis is due to an evolutionarily novel reduction (i.e., “mismatch”) in exposure to immune‐priming symbionts, including soil‐transmitted helminths (STHs; parasitic worms) (Rook 2023). Problematically, most prior tests of the OFH have not accounted for species‐specific effects of different soil‐transmitted helminths, overlooking known differences in their impacts on the immune system. To advance work on the OFH, Cepon‐Robins and colleagues use data from Shuar adults living in the UV and CC to examine the species‐specific effects of infection by two common STH species (*Ascaris lumbricoides* [roundworm] and *Trichuris trichiura* [whipworm]) on three distinct circulating biomarkers of infection linked to allergy and autoimmune disease (total immunoglobulin E, interleukin‐6, and c‐reactive protein). Results suggest that *Ascaris* infection may downregulate and *Trichuris* infection upregulate processes of systemic inflammation that are considered central to the etiology of allergy and autoimmune conditions. This work supports a refined model of the OFH, highlighting that STH species have unique evolved effects on human immunity and must be evaluated separately to identify how changes in STH infection and exposure (e.g., due to sanitation‐related changes linked with market integration) shape negative health outcomes. For readers, this study shows how the rich SHLHP dataset can be used to further our understanding of the co‐evolutionary relationships between pathogens and hosts that underly major evolutionary and health‐related hypotheses.

Extending SHLHP work on intestinal infection and child growth and highlighting the SHLHPs commitment to methodological innovation, Pfaff Nash et al. (Forthcoming) provide what may be the first direct measures of school‐age children's intestinal function among a subsistence‐based population. This short report utilizes the well‐established and popular (in nutrition research) lactulose:mannitol test (L:M test, also known as the “dual‐sugar test”) of intestinal absorption and permeability (Denno et al. 2014). Sugar recoveries in urine were measured using state‐of‐the‐art ultra‐performance liquid chromatograph‐high‐resolution mass spectrometry. Although the sample size is relatively small (*N* = 23) and was drawn from a single CC community, the reported results are shocking. They indicate that rural‐living Shuar children have among the poorest intestinal function on record, with 71%–91% of children categorized as having impaired intestinal function using common L:M cut‐off values. Pfaff Nash et al.'s findings further indicate that both intestinal permeability (i.e., “leaky gut”) and malabsorption are concerns for Shuar children. The indication is that environmental enteric dysfunction (Tickell et al. 2019) is pervasive among the Shuar. As a whole, this work points to impaired intestinal function—through pathways involving both intestinal permeability‐related immune activation and malabsorption‐related energy/nutrient loss—as critical in the etiology of growth faltering among the Shuar (and likely other rural‐living populations, past and present). It is notable that children's growth faltering has been a key issue of concern for Shuar parents since the founding of the SHLHP. This study provides a foundation for future SHLHP and other human biology research to explore the role of intestinal function as a key driver of global health disparities.

Broadening our view of human biological variation and what constitutes “normal” biological function outside of high‐resource, industrialized contexts, Liebert et al. (2025) turn the attention of the special issue to stress and provide an impressive analysis of hypothalamic–pituitary–adrenal (HPA) axis function and related cortisol production among the Shuar. The Shuar experience greater recurrent pathogen exposure (Cepon‐Robins et al. 2014) and more constrained nutrition (Urlacher, Liebert, et al. 2016) than populations such as in the United States, where most HPA‐axis research has taken place. Using a labor‐intensive data collection protocol designed to ensure data quality, Liebert and colleagues collected three saliva samples daily (at waking, 30 min post‐waking, and in the afternoon/evening) on three consecutive days from nearly three‐hundred Shuar participants of all ages in the UV and CC for analysis of salivary cortisol. Their findings demonstrate that the Shuar have extremely low salivary cortisol concentrations (less than 50% of United States reference values among adults) and that Shuar diurnal cortisol patterns have complex, potentially unique, relationships with age, sex, and BMI. Put simply, Shuar HPA‐axis activity does not resemble that of most other populations on record. These data contribute to the cross‐cultural dataset required to understand how critical components of the evolved human stress response present under distinct global socioecological conditions, advancing our understanding of the human stress response in low‐resource, pathogen‐heavy settings, such as those that characterized our recent human evolutionary past.

The work by Barrett et al. (2025) documents important stress‐related health inequities shaped by market integration among the Shuar while again challenging assumptions of “biological normalcy” (Wiley 2023). Specifically, Barrett and colleagues investigate circulating levels of Epstein–Barr virus antibodies (EBV‐Ab, a common immune‐related biomarker of chronic stress) among Shuar and neighboring non‐Indigenous *colonos* of all ages living in 11 UV and CC communities. This is among the first research on EBV‐Ab in a low‐resource, high‐pathogen setting. Results demonstrate that EBV‐Ab levels are higher among Shuar than among *colonos* and vary by factors such as sex, occupation, household level of market integration, and systolic blood pressure. Overall, these findings suggest that EBV‐Ab may be a useful biomarker of the greater socioecological adversity (and presumably chronic stress) experienced by Shuar relative to their non‐Indigenous *colono* neighbors. In contrast to prior studies on EBV‐Ab among higher‐resource, lower‐pathogen populations, however, EBV‐Ab levels among Shuar were not associated with two key predictors: nutritional stress and inflammation. This indicates variation in the relative importance of key socioecological drivers of immune‐related biomarkers of chronic stress between populations. As pointed out by Barrett and colleagues, a key take‐away of this study is that future researchers in human biology must account for population‐specific social and ecological contexts when interpreting biomarkers of stress and health.

Complementing the other included studies on human biological variation and stress response, Tallman et al. (2025) provide an important test of hypothesized links between Shuar self‐reported somatic symptoms of stress (e.g., headaches, body pain, fatigue) and biomarkers of physiological stress (blood pressure and circulating EBV‐Ab concentration). These links are shockingly underexplored in the literature, particularly in non‐industrialized, globally diverse settings, limiting understanding of the universality of such links and how stress shapes human‐wide patterns of health and well‐being. Tallman and colleagues' results from SHLHP data collected from approximately 100 Shuar participants in the UV indicate that adults with the most severe or numerous reported somatic symptoms do indeed have elevated blood pressure and elevated circulating concentrations of EBV‐Ab, indicative of physiological stress response. This work nicely highlights the biocultural, mixed‐methods, and integrated field‐ and lab‐based approach of the SHLHP. Most importantly, the findings demonstrate the consistency of the links between somatic symptoms and physiological biomarkers of chronic stress in a low‐resource context experiencing rapid market integration.

Gildner et al. (2025) turn the focus away from stress to investigate relationships between diurnal salivary testosterone variation, age, and adiposity among Shuar males (aged 12–67 years, from throughout the UV and CC). Once again, the study is framed as an opportunity to test the assumptions of biological normalcy. Current biomedical models of “normal” diurnal testosterone patterns are largely informed by clinical and observational studies in high‐income countries, reflecting a narrow segment of possible ecological conditions known to influence human physiology. Gildner and colleagues’ study contributes data needed to clarify how testosterone production varies across the broader range of human experience, with clear implications for male reproductive health. Results show that testosterone concentrations are shaped by age‐adiposity interactions, suggesting that physical condition and age influence diurnal testosterone variation in complex ways across the life course, potentially due to life history tradeoffs with competing physiological functions. This research supports work conducted among other subsistence populations suggesting that socio‐ecological factors strongly shape testosterone regulation and should therefore be considered when determining what constitutes “normal” testosterone concentrations globally, and within a specific population and context.

Bribiescas et al. (2025) also focus on testosterone and provide the SHLHPs first research on oxidative stress in a novel test for energetic trade‐offs between human male reproductive effort and somatic maintenance effort among rural‐living CC Shuar adults. The hypothesis—informed by findings from non‐human animal models and from human females—that greater male reproductive effort is associated with reduced somatic maintenance effort and correspondingly greater cellular oxidative stress was generally not supported. No clear relationships were detected between Shuar men's urinary testosterone (a marker of investment in reproductive effort), 8‐oxo‐2′‐deoxyguanosine (8‐OHdG, a marker of oxidative stress), and Cu/Zn superoxide dismutase (a marker of antioxidant protection and investment in somatic maintenance effort). Bribiescas et al. offer several potential explanations for this unexpected lack of support for an energetic trade‐off between reproductive and somatic effort, the most promising of which is perhaps that the energetic cost of human male reproductive effort, which is much lower than the cost of female reproduction, is too modest to evoke a detectable trade‐off with somatic maintenance effort among the Shuar. Clarifying the nature of such sex differences in life history trade‐offs is critical for understanding how humans have evolved to manage finite energetic resources. Future research on this topic will benefit from longitudinal study designs that investigate potential trade‐offs at different stages of the lifespan, such as early adulthood when testosterone and reproductive effort peak and late adulthood when senescence of cellular repair mechanisms may exaggerate trade‐offs.

Samsonov et al. (2026) round out the special issue by providing a second analysis of oxidative stress among the Shuar while also returning to the SHLHP focus on market integration. Using a sample of school‐age children drawn from the CC rural and UV peri‐urban extremes of Shuar territory, they investigate, for the first time in any population, the impact of market integration on children's oxidative stress. As they predict, market integration and associated indicators of lower pathogen burden are associated with significantly lower measures of oxidative stress. Peri‐urban Shuar children had, on average, 45% lower oxidative stress index (OSI; the ratio of urinary 8‐OHdG to total antioxidant capacity, reflecting overall oxidative balance) than rural children. Pathogen exposure variables (e.g., unimproved drinking water source), antibody response to macro‐parasites (circulating total Immunoglobulin E), and measured physiological adversity (urinary cortisol concentration) were all exaggerated among rural children and predicted greater OSI and 8‐OHdG across the entire sample. These results suggest that not only is market integration a major driver of global variation in childhood oxidative stress, but also that reduced pathogen burden may be key in this relationship. These are exciting findings. They have implications for understanding how early life adversity and low‐resource contexts drive accelerated biological aging (i.e., oxidative stress‐driven senescence) and poor later life health. They also provide insight into the potential energetic factors (i.e., trade‐offs between oxidative defenses and childhood growth) involved in selection for the remarkably slow human life history pattern.

## 4 Conclusions and the Next 20 Years of the SHLHP

This special issue of the *American Journal of Human Biology* showcases the past 20 years of the SHLHP and its continuing contributions to our understandings of human biology, life history, and health. The 10 included papers span much of the project's intellectual breadth and capitalize on many of its strengths. To advance understanding on how market integration impacts Shuar health and well‐being (project focus #1), for example, many of the included papers capitalize on the wide range and nuance of market integration captured by the SHLHP dataset—from the most isolated rural Shuar households in the CC to the most market‐integrated Shuar households in market centers in the UV. In similar fashion, to better define how lifetime phenotype and health are shaped by adaptive energy allocation between competing life tasks (project focus #2), nearly all the included papers take advantage of the SHLHP's established evolutionary, mixed‐methods approach, often incorporating innovative minimally invasive biomarker analyses in project members’ labs. In the broadest sense, the findings of the included papers reinforce and expand key themes in the contributions of the SHLHP over the past two decades. This is perhaps most notable in documenting the complex, heterogenous nature of market integration on phenotype/health, identifying the role of energetic trade‐offs in generating human biological variation, challenging problematic assumptions regarding human biological “normalcy”, and advancing the field through minimally invasive methodological innovation.

We are hopeful that the next 20 years of the SHLHP will be even more impactful and productive than the first 20. Given the pace of market integration and lifeway change among the Shuar (not to mention the pace of change in academia and the sciences), it is difficult to forecast what the future will hold. Some plans are clear, however. Topically, the SHLHP will respond to Shuar priorities, ongoing health transitions, and on‐the‐ground realities to address an expanded array of phenotypic/health outcomes and underlying biocultural pathways. Much of this work will center on the rapidly accelerating Shuar epidemiological and nutrition transition. It will include new or intensified focus on topics such as obesity, inflammaging, gut dysfunction, bone health, longevity, and food/water insecurity, all of which are emerging concerns for Shuar participants. This work will be supported by an effort to increasingly leverage the project's rich long‐term dataset to perform analyses utilizing life course (e.g., developmental plasticity, resilience) and structural (e.g., infrastructure neglect, racism) frameworks. Through ongoing standardization of human biology datasets, the SHLHP will also increasingly engage in cross‐cultural comparative research to facilitate broader inferences in human biology and beyond.

Importantly, as a project, the SHLHP will continue over the next 20 years to critically reflect on its impact and to prioritize long‐term community investment, local collaboration, and participant benefit. Through a growing number of partnerships, emphasis is increasingly being placed on culturally grounded capacity‐building efforts (e.g., local education and training) to provide maximum benefit to the Shuar and to foster a diverse and local future project leadership. Relationships with local policymakers and other stakeholders are also being strengthened, with outputs being developed in Spanish to maximize impact. A remaining limitation and challenge for the SHLHP is an overreliance on Western researchers and perspectives. As researchers, the SHLHP team recognizes that it is critical to acknowledge how our own cultures, experiences, beliefs, and academic training have shaped our interactions with the Shuar and our engagement with collected data. The project provides considerable opportunity to learn from the Shuar and to expand perspectives through deep local engagement. This includes, for example, the opportunity to incorporate long‐held local conceptions of health and the human body into the project's study design and engagement strategy. As discussed in greater detail elsewhere (Snodgrass et al. Forthcoming), overarching priorities for the SHLHP will remain incorporating Shuar voices at every stage of the research process and being a responsible steward and protector of entrusted Shuar data. It is our hope that this commitment will ensure continued, impactful collaboration for decades to come.

## Ethics Statement

The authors have nothing to report.

## Conflicts of Interest

The authors declare no conflicts of interest.

## Data Availability

Data sharing not applicable to this article. Requests for data used in primary SHLHP papers can be requested through the project's online Data Request Form (https://www.shuarproject.org/data‐sharing).
